# A Case of Robotic Cholecystectomy in a Patient With Decompensated Cirrhosis and Portal Hypertension

**DOI:** 10.7759/cureus.68315

**Published:** 2024-08-31

**Authors:** Matthew J Li, Kelsey R Sprinkles, Mohamed Elfedaly, Basem Soliman

**Affiliations:** 1 Department of Surgery, Texas Tech University Health Sciences Center, Amarillo, USA

**Keywords:** liver cirrhosis, robotic-assisted surgery, minimally invasive surgery, portal hypertension, decompensated liver cirrhosis, severe acute cholecystitis

## Abstract

In patients with liver cirrhosis, approximately one-third experience pigmented cholelithiasis. In parallel to this, cirrhotics consequently encounter a greater prevalence of acute cholecystitis. Traditionally, the definitive treatment for acute cholecystitis in non-cirrhotic patients is cholecystectomy. However, decompensated cirrhosis and portal hypertension pose a surgical challenge, as these comorbidities increase the risk of postoperative complications such as bleeding, infection, and multi-organ failure. Therefore, it is of utmost importance to consider patient risk factors, anatomy, and acuity of patient cholecystitis on an individual basis and develop a surgical (or non-surgical) plan that minimizes risk to patients with decompensated cirrhosis and portal hypertension. We present the management strategies of a case of a 50-year-old male who presents with a history of decompensated liver cirrhosis and portal hypertension complicated by acute cholecystitis. Upon initial presentation, he was critically ill, and a percutaneous cholecystostomy tube was placed for management and the patient was instructed to follow up in the clinic. Then, the patient later returned to the emergency department with a fever, UTI, and sepsis. At that time, his cholecystostomy tube continued to have bilious drainage and he had tenderness in the right upper quadrant. The decision was made to proceed with surgery. Because of his significant comorbid conditions and underlying cirrhosis, surgery posed an increased risk. For this patient, it was especially important to evaluate the risk of complications and the decision of open vs laparoscopic cholecystectomy. In this patient, robotic-assisted laparoscopic cholecystectomy was eventually performed. Due to the patient’s hepatomegaly, splenomegaly, and portal hypertension, special consideration was needed for trocar placement. In this case, we aim to exemplify that is of utmost importance to consider patient anatomy by using imaging and marking organ borders to inform trocar placement as part of the surgical approach.

## Introduction

Cirrhosis is a chronic disease of the liver characterized by hepatic fibrosis, remodeling, and distortion of vasculature that results in increased intrahepatic vascular resistance [1]. In decompensated cirrhosis, portal hypertension develops as a result of increased vascular resistance and leads to greater systemic effects, manifesting as symptoms such as palmar erythema, spider angiomas, ascites, hepatic encephalopathy, porto-systemic collaterals, and acute variceal bleed [2]. In patients with liver cirrhosis, approximately one-third experience pigmented cholelithiasis due to gallbladder hypomobility, increased secretion of unconjugated bilirubin, and decreased bile acid secretion [3]. Ultimately, a greater incidence of cholelithiasis leads to an increased incidence of gallbladder pathology such as acute cholecystitis, 47% in cirrhotic patients and 14.7% in non-cirrhotic patients [4]. Traditionally, the definitive treatment for acute cholecystitis in non-cirrhotic patients is cholecystectomy. However, decompensated cirrhosis poses a surgical challenge, as these comorbidities increase the risk of postoperative complications, such as bleeding, infection, and multi-organ failure [5]. It is also important to consider cases with complicated anatomy or other risk factors such as obesity and diabetes in deciding surgical approaches (open vs. laparoscopic) [6]. Therefore, it is of utmost importance to consider patient risk factors, anatomy, and acuity of patient cholecystitis on an individual basis and develop a surgical (or non-surgical) plan that minimizes risk to patients with decompensated cirrhosis and portal hypertension.

We present the management strategies of a case of a 50-year-old male who presents with a history of decompensated liver cirrhosis and portal hypertension complicated by acute cholecystitis.

## Case presentation

**Figure 1:**
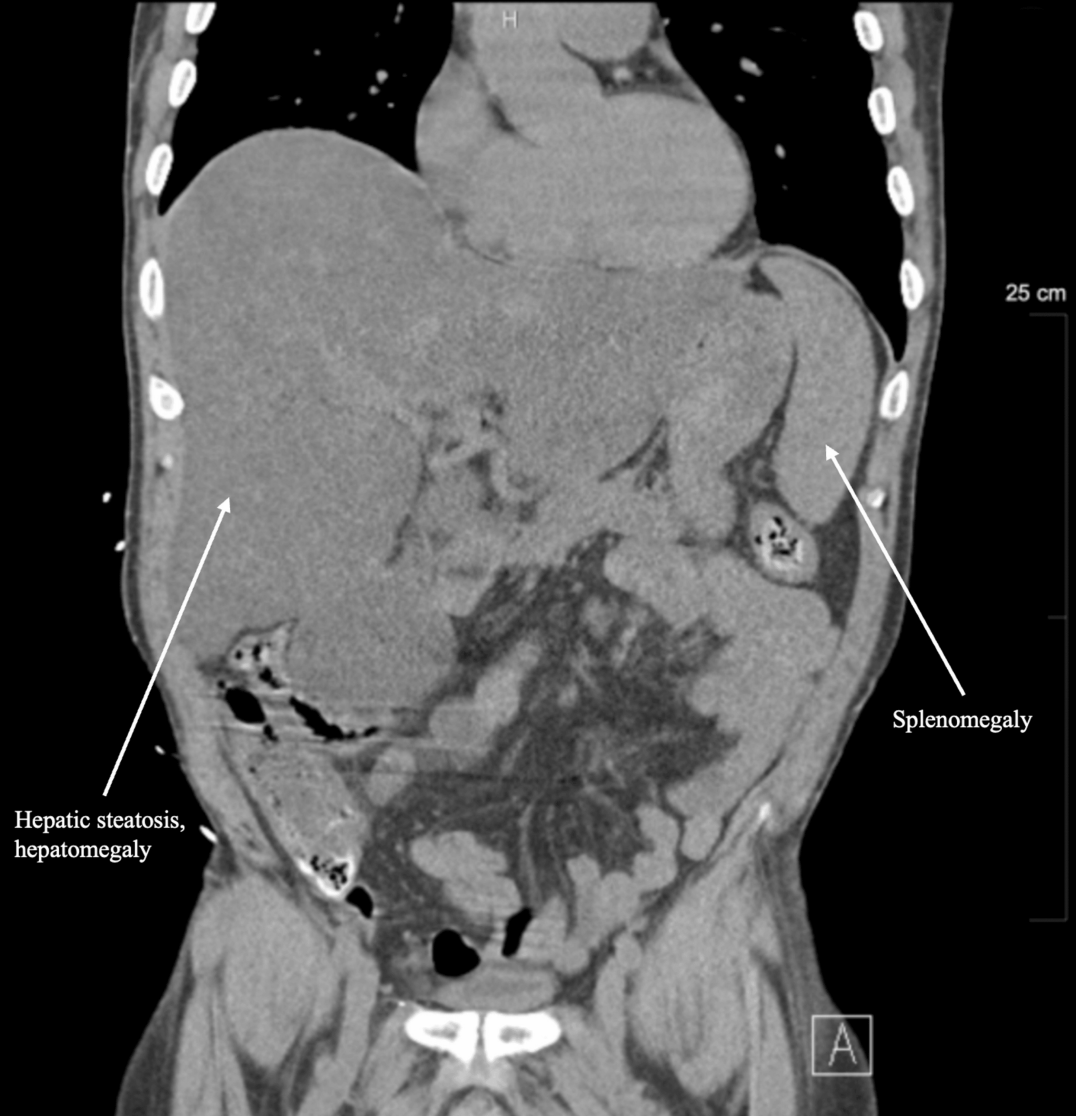
Non-contrast CT scan of the abdomen and pelvis (hospital day one). Initial non-contrast CT of the abdomen and pelvis demonstrating hepatic steatosis, hepatomegaly, distended gallbladder, and splenomegaly.

**Figure 2:**
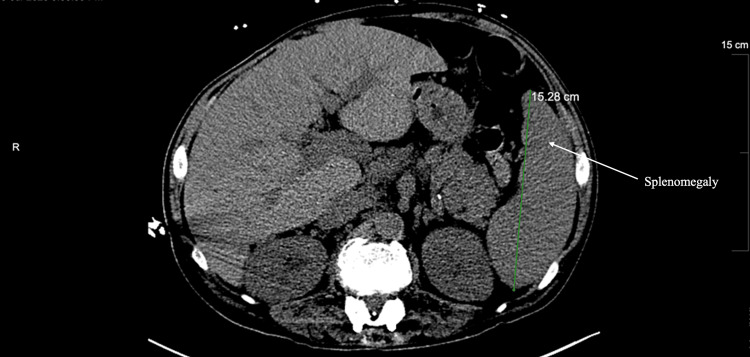
Non-contrast CT of the abdomen and pelvis upon re-presentation. Non-contrast CT of the abdomen and pelvis upon re-presentation demonstrating hepatomegaly, gallbladder decompressed with cholecystostomy tube, and an enlarged spleen measuring 15 cm.

A 50-year-old male, with a medical history of chronic hepatitis C with cirrhosis and a spinal cord injury that requires urinary self-catheterization, presented to the ED with generalized weakness, nausea, and anorexia. He was admitted to the ICU for severe sepsis caused by a UTI and had acute renal failure. The following day, he required intubation and vasopressors. Initial labs showed leukocytosis, thrombocytopenia, elevated total bilirubin, elevated alkaline phosphatase, and increased C-reactive protein (CRP) (Table 1). His blood cultures were positive for Escherichia coli. On day three of his hospital course, there was a concern for gallbladder hydrops and possible acute cholecystitis. Clinically, he had right upper quadrant pain and a positive Murphy’s sign. An ultrasound was performed that showed evidence of acute cholecystitis, including wall thickening, pericholecystic fluid, and positive ultrasound Murphy’s sign. A CT without contrast of the abdomen and pelvis showed severe diffuse hepatic steatosis with hepatomegaly, distended gallbladder, and splenomegaly (Figure 1). The patient continued to have worsening leukocytosis and an elevated total bilirubin. The model for end-stage liver disease (MELD) score and Child-Pugh score were calculated to assess operative risk. The patient received a MELD score of 28 and was classified as Child-Pugh class B. Additionally, the Mayo risk score was developed to serve as a predictor to calculate postoperative surgical mortality risk [7]. The probability of mortality calculated through the Mayo risk score was 29.51% at seven days postoperatively, 76.19% at 30 days postoperatively, 90.15% at 90 days postoperatively, 82.67% at one year postoperatively, and 99.22 % at five years postoperatively. At this point, it was determined that this critically ill patient was at high risk for any type of intervention. Next, a hepatobiliary iminodiacetic acid (HIDA) scan was performed on hospital day three and demonstrated nonvisualization of the gallbladder out to 60 minutes, patent common bile duct, and delayed hepatic uptake and excretion of radiotracer, suggesting underlying hepatic dysfunction. The total bilirubin and LFTs continued to increase. An MRCP was performed on hospital day five to evaluate for possible obstruction, and it demonstrated a massively dilated gallbladder, a mildly dilated common bile duct, but no intrahepatic biliary channel dilation. While the patient initially presented with acute calculous cholecystitis, treatment with antibiotics contributed to decreased inflammation and some symptom control. Due to the patient’s continued critically ill state, a percutaneous cholecystostomy tube was placed, and the patient was instructed to follow up in the clinic. The plan was to keep the cholecystostomy tube in place for two to four weeks. On hospital day 15, the patient’s septic shock had resolved, and the patient was discharged with the cholecystostomy tube in place. Then, 10 days later, he returned to the emergency department with a fever, UTI, and sepsis. At that time, his cholecystostomy tube continued to have bilious drainage of 100-200 mL per 24 hours, and he had tenderness in the right upper quadrant. A CT of the abdomen and pelvis showed an enlarged liver, an enlarged spleen measuring 15 cm, and a gallbladder decompressed with a cholecystostomy tube in its expected position (Figure 2).

**Table 1:** Select laboratory values on hospital day one since initial presentation.

Lab Values	Patient Values	Reference Ranges
Hemoglobin (g/dL)	11.4	14.0-18.0
Platelets (K/uL)	41	130-400
WBC (K/uL)	15.5	4.8-10.8
CRP (mg/dL)	31.920	< 1.000
Albumin (gm/dL)	2.7	3.5-5.7
Bilirubin, Total (mg/dL)	1.4	0.3-1.0
Alkaline Phosphatase (U/L)	148	34-104
ALT (U/L)	41	7-52
AST (U/L)	60	13-39

**Figure 3:**
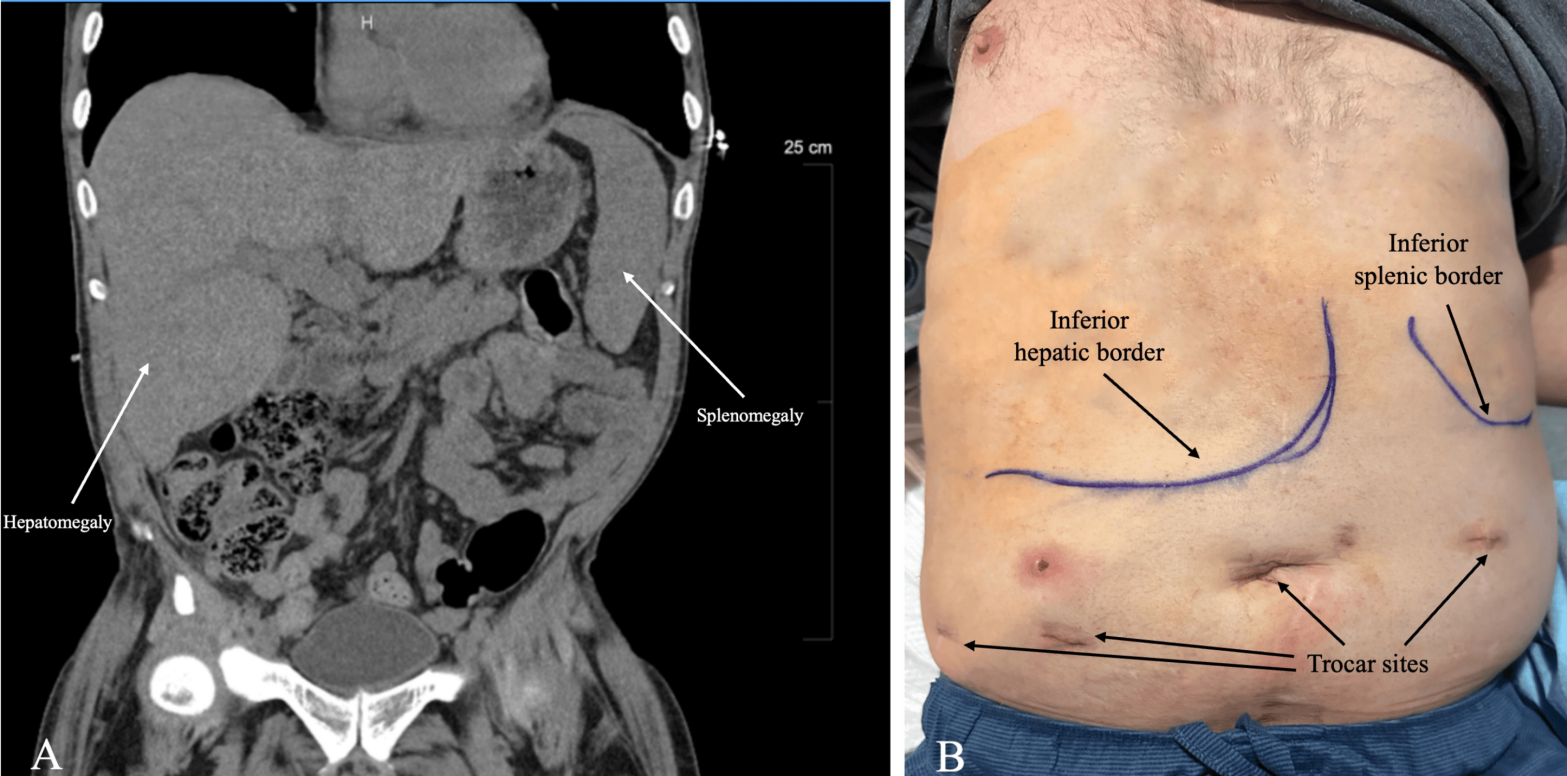
CT-guided preoperative organ marking for safe trocar placement. 3A) Coronal CT of the abdomen and pelvis used to delineate lower organ borders. 3B) Organ borders marked preoperatively using CT from 3A as a guide.

At that time, the decision was made to proceed with robotic-assisted laparoscopic cholecystectomy. After obtaining informed consent, the patient was taken to the operating room for surgery. Due to the patient’s hepatomegaly, splenomegaly, and portal hypertension, special consideration was needed for trocar placement. The patient’s CT image was used to delineate the lower borders of the liver and spleen with a marking pen (Figure 3A, Figure 3B). Trocar placement was determined by the organ borders. Access was obtained via a 5 mm Optiview camera, and the trocars were placed in the periumbilical region and, laterally, both left and right. Note that this is a more inferior position relative to typical placement in robotic-assisted cholecystectomies. Attention was then directed to the right upper quadrant. The patient’s gallbladder was contracted. Additionally, the gallbladder wall was thickened with cholelithiasis, the cystic duct was dilated and shortened, and the liver surface was grossly nodular with signs of advanced cirrhosis. Those findings in conjunction with the enlarged liver provided limited space. The cholecystectomy was performed. There was no evidence of bleeding or leakage of bile at the end of the case. Hemostasis was achieved in the liver bed. The patient tolerated the procedure well, and there were no complications. He was transferred to the recovery room and was stable.

The patient continued to tolerate therapies and was able to advance his diet on postoperative day one. His postoperative hospital course was uneventful, and the patient was discharged seven days later. He later followed up in the outpatient general surgery clinic without nausea, vomiting, fever, or other complications. He was instructed to follow up again in the outpatient general surgery clinic after one year.

## Discussion

Acute cholecystitis is a common surgical pathology. It affects approximately 200,000 people in the US each year [8]. However, in patients with significant comorbidities, surgical planning and decision-making often become exponentially more difficult. Therefore, it is important to contemplate patient status and operative risk in the formation of a treatment plan.

When a patient has significant comorbidities and an underlying diagnosis of cirrhosis, this influences the decision to perform surgery in addition to the operative approach [9]. It is important to evaluate the perioperative and postoperative risks in those who are surgical candidates for cholecystectomy with additional conditions, such as decompensated cirrhosis and portal hypertension. Cirrhosis and its possible systemic complications contribute to increased conversion to open surgery, possibly due to the liver anatomy changes and parenchymal fibrosis that are present in these patients. Patients undergoing cholecystectomy with cirrhosis may have increased operative duration. Additionally, patients presenting with an acute surgical pathology have an increased risk for intraoperative bleeding, contributing to the rationale that a more conservative, less invasive, therapeutic option may be chosen [10]. Furthermore, understanding the level of severity of cirrhosis is critical when deciding if a patient is a surgical candidate. Firstly, a patient’s presentation can be categorized as compensated or decompensated cirrhosis. Then, the severity can be further evaluated using the Child-Turcotte-Pugh (CTP) and the model for end-stage liver disease (MELD) scores. Patients who are classified as Child C would pose a high surgical risk. As was chosen for our patient, a cholecystostomy tube is an alternative in these types of high-risk patients [11].

Abdominal surgeries carry inherent risks, particularly when it comes to post-operative mortality. To mitigate these risks, healthcare professionals rely on two essential tools: the MELD score and the Child-Pugh score [12]. Although both MELD and Child-Pugh scores originated first to predict postoperative mortality after transjugular intrahepatic portosystemic shunt (TIPS) and surgical shunt procedures, respectively, both scores have been validated through various studies to predict post-op mortality after emergency abdominal surgeries. The MELD score evaluates liver function and assesses the risk of mortality for patients with end-stage liver disease. Based on three primary variables, namely, serum bilirubin, creatinine, and international normalized ratio (INR), the MELD score categorizes patients into three groups: low (≤ 9), medium (10-19), and high (≥ 20) risk. Higher MELD scores indicate poorer liver function and a higher likelihood of post-operative mortality. The Child-Pugh score considers various clinical and laboratory factors to evaluate liver function and disease severity. Classifying patients into three categories, namely, A (5-6 points), B (7-9 points), and C (10-15 points), the Child-Pugh score provides a more comprehensive assessment than MELD alone. A higher Child-Pugh score corresponds to a higher risk of postoperative mortality. Studies have consistently demonstrated the significance of MELD and Child-Pugh scores in predicting postoperative mortality for abdominal surgeries. Patients with higher MELD or Child-Pugh scores face a greater risk of complications and mortality after surgery. Specifically, patients with MELD scores of 6-9.9, 10-19.9, 20-29.9, and 30 or higher have predicted 90-day mortality rates of 12.5%, 25%, 37.5%, and 50%, respectively [11]. As mentioned previously, the Mayo risk score is another tool to predict surgical mortality risk for patients with cirrhosis, taking surgery-specific risks (namely, the ASA physical status classification) into account, whereas the MELD and Child-Pugh scores are lacking in this regard [7].

Upon representation to the ER, the patient was in a state of septic shock. In this scenario, it was imperative to operate to achieve source control. However, considerations in surgical approach were necessary to decrease post-operative complications and mortality risk. The question of minimally invasive (robotic/laparoscopic) vs. open cholecystectomy arose. In general, when comparing laparoscopic to open cholecystectomy, one study that reviewed clinical trials demonstrated a significant 17% reduction in postoperative complications, and a significant reduction in the length of hospital stay (3.8 days) [12]. Another study evaluated laparoscopic cholecystectomy compared to open cholecystectomy in patients with cirrhosis and found there to be shorter operative times and reduced length of hospital stays [13]. When comparing robotic vs laparoscopic surgery, robotic consoles offer greater ergonomics, visualization, precision, and dexterity for surgeons [14]. Furthermore, the robotic approach allows surgeons total control of all intra-abdominal instrumentation and camera views, optimizing their own surgical approach. Ultimately, the choice to proceed with robotic cholecystectomy was made due to the stated benefits, especially in this case because of limited visualization and space constraints.

Another consideration in the setting of this patient’s splenomegaly and hepatomegaly was the placement of trocars. Inadvertent injury to the liver, spleen, or portal system could have led to massive hemorrhage in this patient. It was imperative to utilize CT scans to outline the borders of organomegaly in this patient and cautiously place trocars to avoid accidental injury. Indocyanine green (ICG)-enhanced cholangiography was a further measure implemented to reduce the risk of inadvertent injury. This allowed for liver mapping and greater visualization of the cystic duct and common bile duct.

## Conclusions

This case presented a patient with a history of decompensated liver cirrhosis and portal hypertension complicated by acute cholecystitis. Due to posed surgical risks and complications, delayed surgical intervention with temporary management was the optimal strategy. Robotic-assisted laparoscopic cholecystectomy was chosen as the surgical intervention, in part due to limited visualization and space constraints. Robotic-assisted laparoscopic surgery is a recent advancement that offers many benefits for a patient like the one presented in this case. In preparation for surgery, due to the patient’s comorbidities, it was important to use imaging to assess the patient's anatomy and mark the organ borders in the operating room. This is a key nuance to consider in patients presenting similarly to the one in this case. In patients with decompensated cirrhosis, portal hypertension, or splenomegaly, it is important to consider patient anatomy and the extent of their disease to inform trocar placement and surgical approach.
